# Production of G protein‐coupled receptors in an insect‐based cell‐free system

**DOI:** 10.1002/bit.26346

**Published:** 2017-07-03

**Authors:** Andrei Sonnabend, Viola Spahn, Marlitt Stech, Anne Zemella, Christoph Stein, Stefan Kubick

**Affiliations:** ^1^ Fraunhofer Institute for Cell Therapy and Immunology (IZI) Branch Bioanalysis and Bioprocesses Potsdam‐Golm (IZI‐BB) Am Muehlenberg 13 Potsdam 14476 Germany; ^2^ Department of Anesthesiology and Intensive Care Medicine, Charité—Universitätsmedizin Berlin Campus Benjamin Franklin Berlin Germany

**Keywords:** cell‐free protein synthesis, eukaryotic cell‐free system, G protein‐coupled receptor, in vitro translation, insect‐based cell‐free system

## Abstract

The biochemical analysis of human cell membrane proteins remains a challenging task due to the difficulties in producing sufficient quantities of functional protein. G protein‐coupled receptors (GPCRs) represent a main class of membrane proteins and drug targets, which are responsible for a huge number of signaling processes regulating various physiological functions in living cells. To circumvent the current bottlenecks in GPCR studies, we propose the synthesis of GPCRs in eukaryotic cell‐free systems based on extracts generated from insect (*Sf*21) cells. Insect cell lysates harbor the fully active translational and translocational machinery allowing posttranslational modifications, such as glycosylation and phosphorylation of de novo synthesized proteins. Here, we demonstrate the production of several GPCRs in a eukaryotic cell‐free system, performed within a short time and in a cost‐effective manner. We were able to synthesize a variety of GPCRs ranging from 40 to 133 kDa in an insect‐based cell‐free system. Moreover, we have chosen the μ opioid receptor (MOR) as a model protein to analyze the ligand binding affinities of cell‐free synthesized MOR in comparison to MOR expressed in a human cell line by “one‐point” radioligand binding experiments. Biotechnol. Bioeng. 2017;114: 2328–2338. © 2017 The Authors. Biotechnology and Bioengineering Published by Wiley Periodicals, Inc.

**Abbreviations**
CLSM: confocal laser scanning microscopy
CrPV: cricket paralysis virus
ER: endoplasmic reticulum
eYFP: enhanced yellow fluorescent protein
GPCR: G protein coupled receptor
IRES: internal ribosome entry site
mCherry: fluorescent protein
PTM: posttranslational modification
SD: standard deviation
TCA: trichloroacetic acid

## Introduction

G protein‐coupled receptors (GPCRs), consisting of more than 800 members (Grönbladh and Hallberg, 2015), are the largest class of integral membrane proteins. Despite their striking topological similarity, GPCRs respond to a vast number of different extracellular stimuli, such as light, odorants, neurotransmitters, and hormones. Moreover they are the targets of approximately 60% of all drugs (Sarramegn et al., 2006). An essential requirement for GPCR studies is the synthesis of correctly folded and stable receptors in sufficient quantities. To meet the growing demand for preparative amounts of GPCRs, several in vivo synthesis systems based on *Escherichia coli* (*E. coli*), yeast, insect and mammalian cells have been widely used (Chiu et al., 2008; Grisshammer et al., 2005; Lundstrom et al., 2006; Mancia and Hendrickson, 2007; Sarramegna et al., 2003). Nevertheless, the major limitation in functional and structural GPCR studies using cell‐based synthesis is still the production of the desired receptor in sufficient quantity and in its native conformation. The initial experiments and the subsequent data analysis procedure leading to the first crystal structures of GPCRs took several years (Cherezov et al., 2007; Palczewski et al., 2000). Meanwhile improvements in crystallography techniques and protein structure stabilization resulted in new regularly published crystal structures in the last 5 years (Zhang et al., 2015). Up to date, more than 100 crystal structures have been solved. Nevertheless these crystal structures are based on approximately 30 different GPCRs. In conclusion there are still more than 90% of unknown GPCRs structures. Most of these structures might be solved in the future by using appropriate techniques to overcome obstacles in the production of sufficient amounts of GPCRs for crystallization. The expressed GPCRs need to be subjected to solubilization procedures in the presence of detergents prior to purification. This step often reduces the yield of active receptor (Casteleijn et al., 2012). Furthermore, GPCRs might induce a disturbance of downstream signaling cascades or display cytotoxic side effects (Inglese et al., 2007). In this context cell‐free protein synthesis (CFPS) offers a promising alternative to conventional in vivo expression systems. This holds particularly true for difficult‐to‐express proteins as well as posttranslationally modified proteins (Dondapati et al., 2014; Fenz et al., 2013) and toxic proteins (Bechlars et al., 2015). The potential to circumvent cytotoxic effects, together with the open nature of CFPS that enables the simple addition of ligands, co‐factors, and chaperones, are outstanding criteria for the improved synthesis of active GPCRs (Klammt et al., 2007; Schwarz et al., 2008). These advantages have yielded different commercially available cell‐free systems mainly based on *E. coli* and wheat germ extracts (WGE). A major disadvantage of these systems is the required addition of a suitable detergent to solubilize and stabilize de novo synthesized membrane proteins (Bernhard and Tozawa, 2013). Furthermore, many GPCRs require posttranslational modifications (PTMs) such as phosphorylation, palmitoylation, glycosylation, and disulfide bond formation to stabilize their active state and correct folding (Klammt et al., 2004; Merk et al., 2015). Neither *E. coli* nor WGE contain the necessary machinery to ensure complete posttranslational protein processing. In this context, novel eukaryotic lysates represent a promising alternative for the production of active membrane proteins (Dondapati et al., 2014; Quast et al., 2016a). *Spodoptera frugiperda* 21 (*Sf*21) cell lines in particular, are suitable for the preparation of translationally active lysates (Kubick et al., 2009). Due to a mild cell‐disruption procedure, these lysates harbor endogenous microsomes enabling membrane proteins to be co‐translationally translocated into endoplasmic reticulum (ER) derived microsomal structures (Stech et al., 2013). This process is an essential prerequisite for secreted and transmembraneous proteins to undergo PTMs (Mikami et al., 2006; Zeenko et al., 2008).

In this study, we demonstrate the suitability of the *Sf*21‐based eukaryotic cell‐free translation system for the rapid and detergent‐free production of GPCRs in adequate yields. Seven exemplary members of three major GPCR families were produced in this cell‐free system (Table I). The performance of the batch‐based transcription–translation system was optimized with regard to improved synthesis rate and PTM of these receptors.

**Table I:** Summary of GPCRs investigated in this study

GPCR	GPCR family	MM (kDa)	Abbreviation	UniprotKB	PTMs
μ Opioid receptor	Class A	44.5	MOR	P33535 (OPRM_RAT)	5 Phosphorylation sites 5 N‐Glycosylation sites 1 Palmytoylation site 1 Disulfide bond
Metabotropic glutamate receptor 1	Class C	132.4	GRM1	Q13255 (GRM1_HUMAN)	4 N‐Glycosylation sites 5 Disulfide bonds
Glucagon‐like peptide 1 receptor	Class B	53.0	GLP1R	P43220 (GLP1R_HUMAN)	3 N‐Glycosylation sites 4 Disulfide bonds
G‐protein coupled receptor 56	Class B	77.7	GPR56	Q9Y653 (GPR56_HUMAN)	7 N‐Glycosylation sites
Thyrotrophic receptor	Class A	86.8	TSHR	P16473 (TSHR_HUMAN)	6 N‐Glycosylation sites 2 Disulfide bonds
C‐X‐C chemokine receptor type 4	Class A	39.7	CXCR4	P61073 (CXCR4_HUMAN)	3 Sulfation sites 8 Phosphorylation sites 2 N‐Glycosylation sites 1 O‐Glycosylation site 2 Disulfide bonds
C‐X‐C chemokine receptor type 5	Class A	41.9	CXCR5	P32302 (CXCR5_HUMAN)	2 N‐Glycosylation sites 1 Disulfide bond

MM, molecular mass; PTMs, posttranslational modifications.

## Materials and Methods

### Generation of Vectors for Cell‐Free Protein Production

The μ opioid receptor (MOR) encoding DNA sequence (Uniprot accession no. P33535) with N‐terminally fused FLAG epitope was subcloned into the vector pcDNA3.1. Additionally, the same coding sequence was subcloned into the EasyXpress pIX3.0 vector (Qiagen, Hilden, Germany). DNA of Cricket paralysis virus intergenic region internal ribosome entry site (CrPV IGR IRES GenBank accession no. AF218039, nucleotides 6025 to 6216) was synthesized and manufactured by Life Technologies (Darmstadt, Germany) into the pMA vector as described previously (Brödel et al., 2013b). The FLAG‐MOR coding sequence was fused to the DNA sequence of CrPV IGR IRES by a two‐step overlap extension PCR. In the first PCR step, the coding sequence of the CrPV IGR IRES and the gene of interest (MOR) were amplified separately. In the second PCR step, regulatory sequences containing the cloning sites *Eco*RI and *Xho*I were added to the 5′ and 3′ non‐coding regions of the fused CrPV‐FLAG‐MOR (TCF‐MOR) and X‐CrPV‐FLAG‐MOR (NCF‐MOR) templates as described previously (Brödel et al., 2013b). Additionally, in the first and second PCR steps IRES‐specific forward and gene‐specific reverse primer pairs were used to amplify and fuse the individual melittin signal sequence to the gene of interest (NCMF‐MOR). Amplified linear DNA templates were subsequently digested with *Eco*RI and *Xho*I restriction nucleases and the resulting fragments were cloned into the EasyXpress pIX3.0 vector. DNA encoding enhanced yellow fluorescent protein (eYFP), fluorescent protein mCherry, and the fusion products of eYFP/mCherry and MOR (MOR‐eYFP and MOR‐mCherry) were subcloned into EasyXpress pIX3.0 vector (Qiagen).

Nucleotide sequences of cloned constructs were confirmed by DNA sequencing. cDNAs of metabotropic glutamate receptor 1 (GRM1, Uniprot accession no. P33535), glucagon‐like peptide 1 receptor (GLP1R, Uniprot accession no. P43220), G‐protein coupled receptor 56 (GPR56, Uniprot accession no. Q9Y653), thyrotrophic receptor (TSHR, Uniprot accession no. P16473), C‐X‐C chemokine receptor type 4 (CXCR4, Uniprot accession no. P61073), and C‐X‐C chemokine receptor type 5 (CXCR5, Uniprot accession no. P32302) were synthesized and manufactured by Life Technologies into the pMA vector. Synthesized GPCR constructs included regulatory elements and a CrPV IGR IRES sequence upstream of the coding sequence.

### Lysate Preparation Procedure

Eukaryotic cells were grown in well‐controlled fermenters until they reached the exponential growth phase. Insect cells were cultured at 27°C. Cell cultivation was performed using chemically defined, serum‐free media (*Sf*21: Insect‐XPRESS medium, Lonza). Cells were harvested at a density of approximately 4.0 × 10^6^ cells/mL and collected by centrifugation at 200*g* for 5 min. The resulting cell pellets were washed twice and resuspended in a buffer containing 40 mM HEPES‐KOH (pH 7.5), 100 mM NaOAc, and 4 mM DTT. Cells were disrupted mechanically by passing the cell suspension through a 20‐gauge needle using a syringe. Next, the crude cell lysate was centrifuged at 10,000*g* for 10 min in order to remove the nuclei and cell debris. Supernatants were applied to a Sephadex G‐25 column (GE Healthcare, Freiburg, Germany), equilibrated with the above mentioned resuspension buffer, and the elution fractions (1 mL each) with an RNA content above an absorbance of 100 at 260 nm were pooled. Cell lysates were treated with micrococcal nuclease (S7) in order to degrade residual mRNA. In this respect, 10 U/mL S7 nuclease (Roche, Mannheim, Germany) and 1 mM CaCl_2_ were added to the eluate and the reaction mixture was incubated for 2 min at room temperature. The reaction was inactivated by the addition of 6.7 mM EGTA (f. c.). Finally, cell lysates were immediately shock‐frozen in liquid nitrogen and stored at −80°C to preserve maximum activity.

### Cell‐Free Protein Synthesis

Coupled transcription–translation reactions were performed in batch mode. Protein production was mainly operated at 33C in a thermo mixer (Thermomixer comfort, Eppendorf, Hamburg, Germany) with gentle shaking at 500 rpm. Reactions were composed of 40% (v/v) *Sf*21 cell lysate, canonical amino acids (100 μM each), nucleoside triphosphates (1.75 mM ATP, 0.30 mM CTP, 0.30 mM GTP, and 0.30 mM UTP), 60 nM vector DNA, and 1 U/μL T7 RNA‐polymerase (Agilent, Waldbronn, Germany). To monitor protein quality and quantity, reaction mixtures were supplemented with ^14^C‐labeled leucine (specific radioactivity 75.0 dpm/pmol). No template controls (NTC) were prepared in the same way as the samples with the exception of the DNA template which was replaced by RNase‐free water.

### Determination of Protein Yield

Yields of de novo synthesized proteins in translation mixtures were determined by hot trichloroacetic acid (TCA) precipitation followed by liquid scintillation counting as described previously (Brödel et al., 2013a; Stech et al., 2012).

### SDS–PAGE and Autoradiography

Aliquots of 5 μL of *Sf*21 cell‐free reaction mixtures were subjected to cold acetone precipitation. Samples were centrifuged for 10 min at 16,000*g* and 4°C. Protein pellets were resuspended in 20 μL of 1× sample buffer (NuPAGE® LDS Sample Buffer, Life Technologies) and loaded on precast SDS‐PAGE gels (Nu PAGE 10% Bis–Tris gel, Life Technologies). Gels were run in MES SDS buffer for 35 min at 185 V. Subsequently, gels were stained using SimplyBlue Safe Stain (Life Technologies), washed with H_2_O and then dried for 70 min at 70°C (Unigeldryer 3545D, Uniequip, Planegg, Germany). Bands of SeeBlue Plus2 Pre‐Stained Standard (Life Technologies) were labeled using a radioactive marker in order to identify the molecular masses of synthesized target proteins. Finally, radioactively labeled proteins were visualized using a phosphorimager system (Typhoon TRIO+ Imager, GE Healthcare) after a minimum of 2 days of incubation.

### Fluorescence Analysis

Integration of MOR‐eYFP and MOR‐mCherry fusion proteins into microsomal membranes was visualized by confocal laser scanning microscopy (LSM 510, Carl Zeiss, Jena, Germany). Samples were transferred to ibidi slides (μ‐slide, 18 well, Ibidi, Planegg, Germany) and fluorescent proteins were excited at 488 nm (eYFP) and 587 nm (mCherry) using an argon laser. Emission signals were acquired with a long pass filter in the wavelength range above 505 nm.

### Cell Culture of HEK 293 Cells and Radio Ligand Binding Assay

Human embryonic kidney (HEK) 293 cells stably expressing rat MOR were maintained in Dulbecco's Modified Eagle Medium (Sigma–Aldrich, Steinheim, Germany) supplemented with 10% fetal bovine serum, 1% penicillin/streptomycin and 0.1 mg/mL geneticin (Biochrome, Berlin, Germany) at 37°C and 5% CO_2_ in a cell incubator. They were passaged 1:3–1:10 every second to third day depending on their confluency. For binding experiments MOR expressing cells were cultured in flasks with a growth area of 175 cm^2^. Cells were washed with ice‐cold Trizma (50 mM, pH 7.4) (Sigma–Aldrich), scraped off with a cell scraper, homogenized and centrifuged twice at 42.000*g* for 20 min at 4°C as described previously (Busch‐Dienstfertig et al., 2013; Spahn et al., 2013, 2014). Protein concentration was determined using the Bradford method (Bradford, 1976). Binding experiments with labeled MOR ligands [^3^H]‐D‐Ala2, N‐MePhe4, Gly‐ol]‐enkephalin (DAMGO) and—[^3^H]‐naloxone (NLX), respectively, were carried out according to a modified protocol (Busch‐Dienstfertig et al., 2013). Briefly, 100 μg of cell membranes were prepared and incubated for 90 min in assay buffer (50 mM Trizma, pH 7.4) with increasing doses of [^3^H]‐DAMGO (0.5–16 nM) (47.1 Ci/mmol) and [^3^H]‐NLX (0.9–15 nM) (58.2 Ci/mmol) (Perkin Elmer, Waltham, MA), respectively, for saturation binding studies or with 4 nM [^3^H]‐DAMGO for single point binding studies in the absence or presence of 10 μM unlabeled NLX to determine nonspecific binding. The conversion of counts per min (cpm) to fmol/mg total protein was realized using the following equation: (cpm × 100)/(counter efficacy × 2.2 × specific activity of [^3^H]‐DAMGO (or [^3^H]‐NLX) × total protein amount in mg).

**Figure 1:**
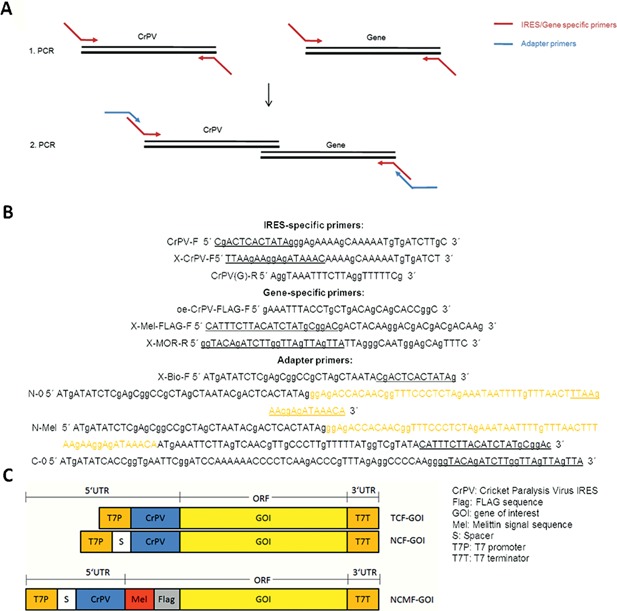
Schematic overview of strategies for the generation of linear DNA templates. (A) Amplification of the target gene was initially realized using gene‐specific and IRES‐specific primers, respectively (red). Adapter primers (blue) were applied in a second PCR step to link the amplified target gene to regulatory sequences, signal sequences and sequences encoding affinity tags. (B) Sequence of adapter primers, genespecific, and IRES‐specific primers. Overlapping sequences are underlined and the spacer (S) sequence is highlighted in orange. (C) Schematic overview of the generated linear DNA templates harboring regulatory elements.

Samples of cell‐free synthesized MOR were centrifuged at 16,000*g* and 4°C for 10 min. Thereafter, they were either directly dissolved in binding assay buffer, or pretreated with 500 mM sorbitol, 0.05% or 1% Dodecyl‐β‐D Maltosid (DDM) for 15 min on ice, followed by centrifugation at 16,000*g* and 4°C for 10 min and dissolving in assay buffer. Binding experiments were carried out the same way as described for HEK 293 MOR cells (Fig. 1).

## Results

### Optimization of Cell‐Free Protein Synthesis Reaction Conditions

**Figure 2:**
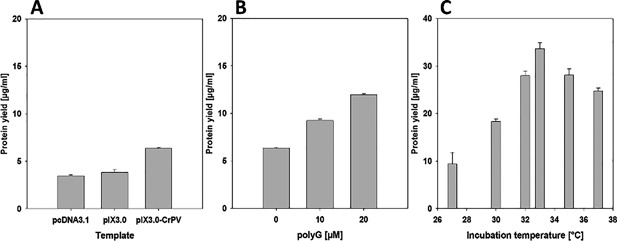
Evaluation of cell‐free reaction conditions. (A) Comparative analysis of MOR encoding synthesis vectors. (B) Influence of polyG supplementation on MOR synthesis levels with CrPV IGR IRES. (C) Influence of 20 µM polyG and temperature on cell‐free synthesized MOR using pIX3.0 vector.

Cell‐free protein synthesis was performed in coupled transcription–translation reactions. Initial reaction conditions were optimized for the synthesis of ^14^C‐leucine‐labeled MOR in the batch‐based *Sf*21 cell‐free system (Fig. 2). Protein synthesis levels were monitored after 2 h of incubation at 27–37°C.

The pcDNA3.1 and EasyXpress pIX3.0 vectors with MOR‐encoding constructs were tested for their performance in the cell‐free reaction (Fig. 2A). Vectors with and without the CrPV IGR IRES element were used to demonstrate the impact of the vector backbone on receptor synthesis (Brödel et al., 2013c). Highest protein yields up to 6.4 μg/mL were monitored using the EasyXpress pIX3.0 vector equipped with the CrPV IGR IRES sequence (Fig. 2A). These pIX3.0 CrPV IGR IRES (GCT) vectors were the starting point for further optimization steps, in particular in terms of the effect of free polyguanylic acid (polyG) and reaction temperature on protein translation.

PolyG supplementation significantly enhanced translation of MOR at any concentration tested (Fig. 2B). Highest protein yields were obtained at a final concentration of 20 μM polyG (∼12 μg/mL). In the presence of 20 μM polyG, the optimum incubation temperature was discovered to be 33°C, whereas any further increase in temperature resulted in reduction of synthesis levels (Fig. 2C). The synergistic effect of optimization steps increased protein yields from 6.4 to 33.7 μg/mL. These results demonstrate a five‐fold increase of de novo synthesized ^14^C‐leucine‐labeled MOR compared to the initial situation.

### Synthesis of Different GPCRs in *Sf*21‐Based Cell‐Free System

**Figure 3:**
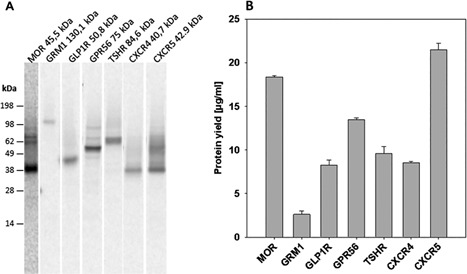
Synthesis of different GPCRs in Sf21 cell lysates. Cell‐free protein synthesis was performed in the presence of 14C‐leucine. (A) Qualitative analysis of cell‐free synthesized GPCRs by SDS‐PAGE and autoradiography. MOR, µ opioid receptor; GRM1, metabotropic glutamate receptor 1; GLP1R, glucagon‐like peptide 1 receptor; GPR56, G‐protein coupled receptor 56; TSHR,thyrotrophic receptor; CXCR4, C‐X‐C chemokine receptor type 4; CXCR5, C‐X‐C chemokine receptor type 5. (B) Quantitative analysis of ^14^C labeled receptors by liquid scintillation counting.

The initial optimization process was followed by expanding the use of the improved cell‐free system for the synthesis of different GPCRs. Six other receptors were synthesized in vitro under optimized reaction conditions (Fig. 3). The integrity and appropriate size of ^14^C‐labeled receptors was visualized by autoradiography after gel electrophoresis (Fig. 3A). Table I illustrates GPCRs investigated in this study with different PTMs and molecular mass (MM) in the range of 40–133 kDa. The detected apparent MM of the MOR, the metabotropic glutamate receptor 1 (GRM1), the C‐X‐C chemokine receptor type 4 (CXCR4), and the C‐X‐C chemokine receptor type 5 (CXCR5) corresponds to the expected MM of the individual cell‐free synthesized protein. In case of glucagon‐like peptide 1 receptor (GLPR1), G‐protein coupled receptor 56 (GPR56), and thyrotrophic receptor (TSHR) the apparent mass is significantly lower than the expected MM. The migration through the gel might be altered due to the interaction between hydrophobic transmembrane domains with SDS: these interactions might result in a lower apparent molecular mass (Rath et al., 2009). The upper bands in the lanes of MOR and CXCR5 might represent multimers. It is known for both receptors, that a multimerization is possible (Al‐Hasani and Bruchas, 2011; Salanga et al., 2009). In addition, the corresponding TCA precipitation data demonstrate that the yields are in the range of 3–22 μg protein per mL reaction within 2 h of incubation.

### Microsomal Integration and Posttranslational Modification of GPCRs

**Figure 4:**
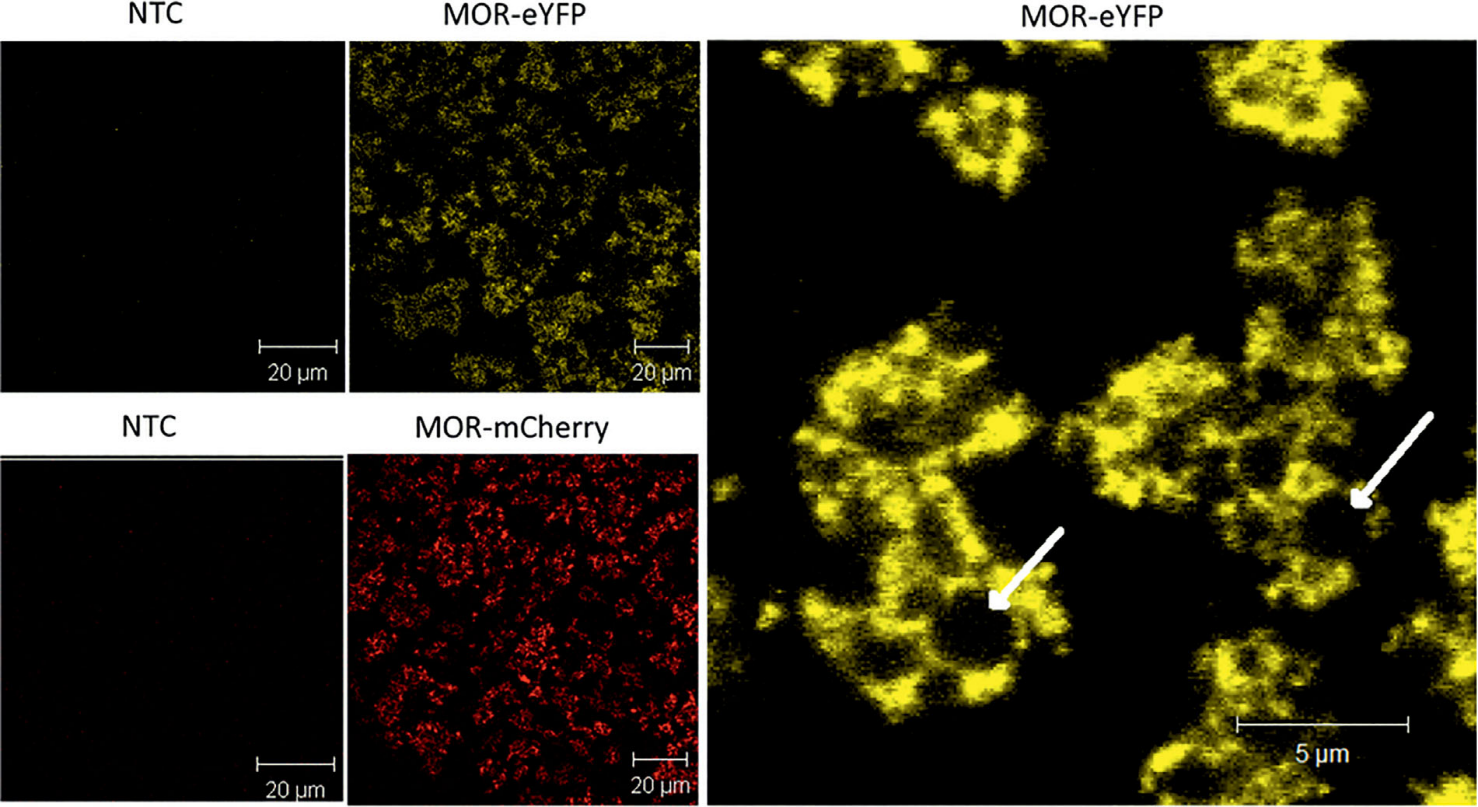
Fluorescence analysis of eYFP/mCherry‐tagged receptors synthesized in the coupled Sf21 cell‐free system using CLSM (LSM 510 Meta, Carl Zeiss). Fluorescent insect vesicles in the vesicular fraction (VF) indicate efficient incorporation of MOR into microsomal membranes. NTC: no template control. Samples were excited at 488 nm using an argon laser and fluorescence emission was recorded with a longpass filter in the wavelength range above 505 nm. Arrows exemplarily indicate a microsomal vesicle harboring the fluorescent target protein.

In order to analyze the integration of cell‐free produced MOR‐eYFP and MOR‐mCherry fusion proteins into the ER‐derived microsomes, samples were analyzed by confocal laser scanning microscopy (CLSM, LSM 510 Meta, Carl Zeiss). Integration of receptors into the microsomal membrane of insect vesicles was visualized by fluorescence analysis of the vesicular fraction (VF), since it is possible to separate insect vesicles containing fluorescent fusion protein from the cytosolic fraction of the lysate by centrifugation (Fig. 4).

### Ligand Binding Properties of Cell‐Free Synthesized MOR

**Figure 5:**
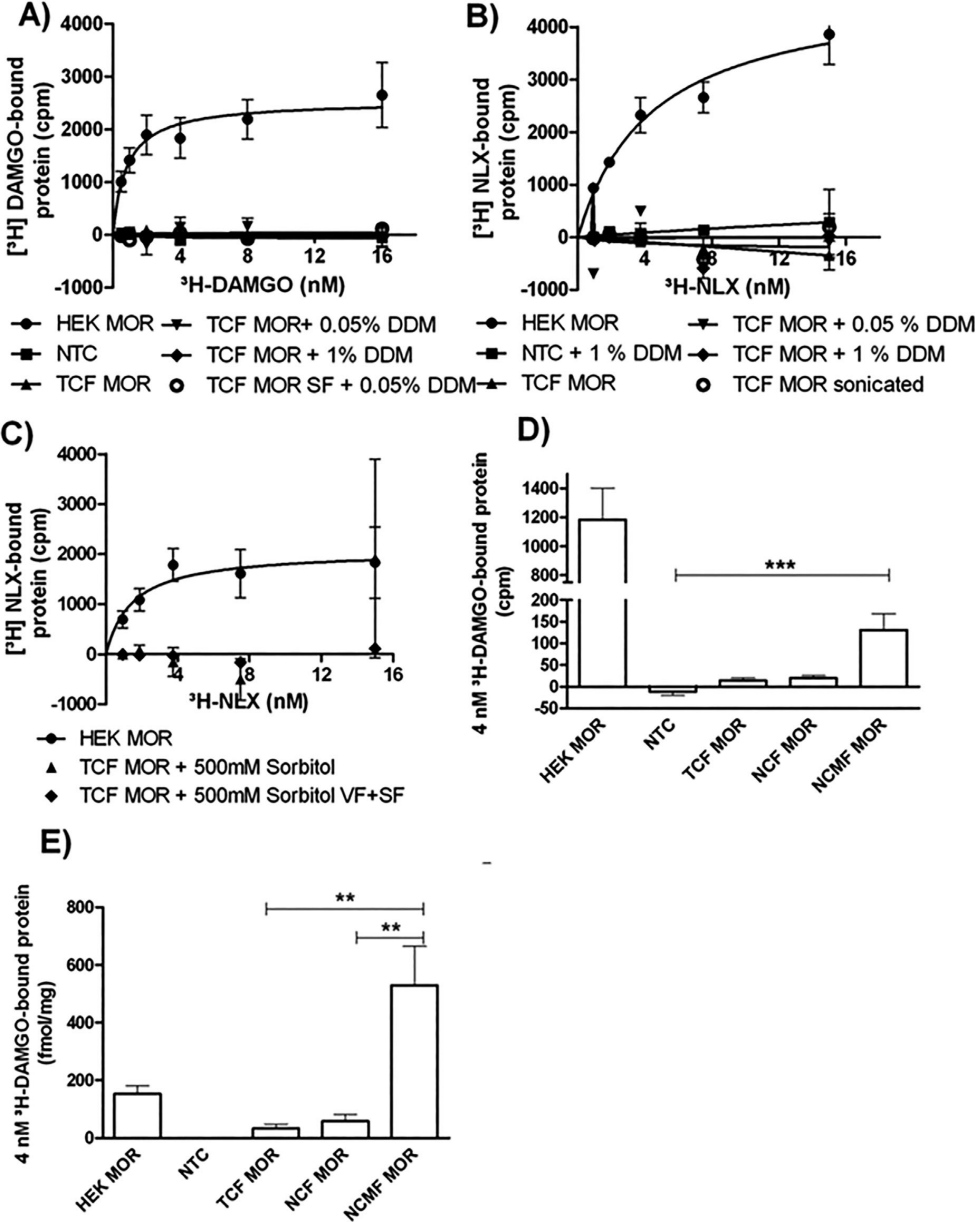
Characterization of the binding properties of [3H] DAMGO and [^3^H]‐Naloxone (NLX) to MOR expressed in HEK 293 cells and MOR expressed in a cell‐free synthesis system. (A) [^3^H]‐DAMGO saturation binding experiments of HEK MOR and TCF‐MOR in presence of different detergents. (B and C) [^3^H]‐NLX saturation binding experiments of HEK MOR and TCF‐MOR in the presence of different detergents. (D) [^3^H]‐DAMGO single point binding experiment of HEK MOR, TCF‐MOR, NCF‐MOR, and NCMF‐MOR (*n* = 6) presented as counts per minute (cpm). (E) [^3^H]‐DAMGO single point binding experiment of HEK MOR, TCF‐MOR, NCFMOR, and NCMF‐MOR (*n* = 6) presented as fmol/mg total protein.

To investigate the ligand binding properties of cell‐free synthesized MOR in comparison to MOR expressed in HEK 293 cells, we performed radioligand binding assays. Using 100 μg total protein per sample of stable MOR HEK 293 cells (HEK MOR) and 54 ng–2.16 μg per sample of pure cell‐free synthesized MOR (TCF‐MOR, Fig. 5A). In contrast to HEK MOR, TCF‐MOR did not show a specific [^3^H]‐DAMGO binding signal. We assumed that the ligand binding domains of MOR could have been located inside the insect vesicles. Therefore, we tested different detergents to perforate the vesicles and render the binding pockets accessible for the ligands. Using 0.05 and 1% of the mild and non‐ionic detergent DDM, we did not detect any specific binding signal in comparison to the no template control (NTC) (Fig. 5A; 2‐way ANOVA with Bonferroni's post hoc test, *P* > 0.05). In addition, we checked the supernatant fraction of cell‐free translation mixtures, where approximately 50% of the total protein concentration was detected (data not shown). However, we did not measure binding of [^3^H]‐DAMGO or [^3^H]‐NLX (Fig. 5B and C) under the same conditions. Cell‐free synthesized MOR was treated either with 0.05 and 1% DDM; 500 mM sorbitol or phosphate buffered saline. Again, we were not able to measure specific NLX‐binding. To further optimize the translocation of MOR into the membrane of ER‐derived vesicles, we added a melittin signal sequence N‐terminally to our constructs (NCMF‐MOR). Alternatives to saturation binding are “one‐point” radioligand binding experiments, which dramatically reduce the required amount of protein. Initial one‐point binding experiments using NCMF and the conventional constructs without signal sequence TCF in the presence or absence of the detergents DDM, sorbitol and Brij 35 indicated similar specific [^3^H]–DAMGO binding to cell‐free synthesized NCMF‐MOR (Fig. 5D). Therefore, we performed the following experiments without addition of detergents. The signal of 4 nM [^3^H]–DAMGO‐bound protein (in counts per minute) of NCMF‐MOR was significantly higher compared to TCF‐MOR and NCF‐MOR (Kruskal Wallis test with Dunn's post hoc test, *P* < 0.001, ***). Normalization to the respective applied protein concentrations showed a tendency of higher 4 nM [^3^H]–DAMGO‐bound protein (fmol/mg) of NCMF‐MOR compared to MOR expressed in HEK 293 cells (Fig. 5E). The 4 nM [^3^H]–DAMGO‐bound protein between the cell‐free synthesized MOR constructs differed significantly (Kruskal–Wallis test with Dunn's post hoc test, *P* < 0.01, **).

## Discussion

In summary, our study demonstrates the ability of an *Sf*21‐based eukaryotic cell‐free translation system for rapid, and detergent‐free production of 7 different GPCRs. The performance of the batch‐based transcription–translation system has been optimized in regard to GPCR synthesis rate of the receptors. The use of the EasyXpress pIX3.0 vector equipped with the CrPV IGR IRES sequence showed enhanced protein yields compared to the pcDNA 3.1 vectors and the EasyXpress pIX3.0 vector without IRES. The supplementation of polyG significantly enhanced the translation efficiency of MOR. Furthermore, we demonstrated microsomal integration of MOR‐eYFP and MOR‐mCherry. In addition to the successful cell‐free synthesis of different GPCRs, we showed for the first time, using MOR as a model protein, that DAMGO, a protypical MOR ligand, binds to cell‐free synthesized MOR in a comparable manner to MOR expressed in HEK 293 cells.

The investigation of proteins in the context of their function and structure requires their synthesis in suitable quantities. So far, three strategies are commonly used to produce proteins: chemical synthesis (peptides), in vivo synthesis in host cells, and cell‐free protein synthesis (Endo and Sawasaki, 2006). The last mentioned strategy provides substantial advantages since the synthesis is not limited by the length of the synthesized peptide or host toxicity, aggregation, misfolding or degradation (Gagoski et al., 2015). There are several cell‐free systems based on different extracts, for example, *E. coli*, rabbit reticulocytes and wheat germ extracts. So far, these systems present numerous drawbacks regarding correct posttranslational modifications or sufficient protein yields (Endo and Sawasaki, 2006; Zheng et al., 2014). However, for the investigation of structural and functional characteristics of transmembrane proteins like GPCRs, it is of importance to produce them in sufficient amounts displaying their native posttranslational modifications. Investigations on a non‐glycosylated human MOR revealed a decreased overall stability of the receptor (Huang et al., 2012). These findings have been observed for various GPCRs. In contrast to the effects on the stability of MOR, a reduced N‐glycosylation displayed no influence on binding‐affinities of opioid ligands (Huang et al., 2012). A similar effect has been observed in MOR without palmitoylation. Whereas the palmitoylation does not affect the binding of tested ligands, the ligand‐induced receptor signaling, dimerization and G protein‐coupling was slightly impaired (Zheng et al., 2012). This finding seems to be quite obvious since the binding pocket is located in transmembrane domains that might not be affected by N‐glycosylation and palmitoylation. Nevertheless depending on the expression system, different glycosylation patterns are observed. As mentioned above, inappropriate glycosylation might result in reduced activity, limited half‐life and immunogenicity (Khan et al., 2016). Usually high mannose type and paucimannose N‐glycans are built in insect cells whereas complex‐ and hybrid‐N‐glycans are synthesized in human cells. Moreover a α1.3‐fucose is often added to the core of the glycan that might result in immunogenic reactions. Sialylation is usually not observed in insect cells. Similar problems can often be found in cell lines based on baby hamster kidneys and Chinese hamster ovary (CHO). In CHO cells, a different form of sialic acid is usually utilized and nonhuman mammalian cells often display a different linkage between oligosaccharides which might be immunogenic as well (Khan et al., 2016). Recent improvements in cell‐line engineering circumvent most of the mentioned obstacles in mammalian in vivo expression. In eukaryotic cell‐free systems due to the missing Golgi apparatus only core N‐glycosylations can be expected in eukaryotic cell‐free systems. In comparison to in vivo expression systems, a significantly lower heterogeneity of glycan structures can be assumed in cell‐free systems. Nevertheless these alterations might lead to modified protein characteristics. This drawback might be circumvented by the open nature of cell‐free systems that enables the addition of further components (Quast et al., 2015a). In this context the desired glycan structure of the target protein might be built up by using amber suppression techniques in combination with site‐directed click reactions to attach premade sugar moieties to the desired glycoprotein.

In the current study, we used a eukaryotic cell‐free translational system based on *Sf*21 cell lysates and improved protein yields of synthesized MOR by investigating the influence of different vector backbones, the addition of free polyG and the effect of varying reaction temperatures. Recently, we showed that translation initiation in a cap‐independent manner by using DNA templates containing an internal ribosome entry site (IRES) increased production yields in batch based translation systems based on CHO and human cell extracts (Brödel et al., 2013b,2013c). Numerous studies showed that particular IRES sequences from the intergenic region of *Dicistroviridae* can bypass the process of translation initiation, which is one of the limiting steps in cell‐free protein synthesis (Mikami et al., 2006; Zeenko et al., 2008). Additionally, a study using eukaryotic cell‐free systems derived from cultured *Sf*21, CHO and *K562* cells as well as wheat germ identified that IGR IRES from the *Cricket paralysis virus* (CrPV) resulted in higher protein yields compared to IRES sequences from *Israeli acute paralysis virus* or *Taura syndrome virus* (Brödel et al., 2013b,2013c). In the present study, parameters such as ion concentrations, incubation temperature, reaction time, polyG, and CrPV IGR IRES have been investigated extensively. As protein expression in cell‐free systems is sensitive to ions (Jackson, 1991; Kubick et al., 2009) and CrPV IGR IRES‐driven translation functions best at comparatively high quantities of potassium compared to cap‐dependent translation (Cevallos and Sarnow, 2005), the concentrations of potassium and magnesium were adjusted to 120 and 3.4 mM, respectively.

The ability of free polyG to increase the yields in cell‐free systems was demonstrated before in wheat germ extracts by inhibiting RNase activity (Noireaux et al., 2003). Here, we show that polyG also increases protein yields in eukaryotic *Sf*21‐based cell‐free systems. In our system, the optimal reaction temperature of the translation mixture was 33°C which was different compared to previously published studies (Brödel et al., 2013c).

Membrane protein synthesis in insect cell‐free systems has already started over 10 years ago. Although yields for soluble proteins are frequently lower compared to *E.coli* and wheat germ based cell‐free systems, membrane protein synthesis in insect based cell‐free systems has meanwhile reached a comparable level (Shinoda et al., 2016). The insect cell‐free synthesis system initially achieved membrane protein yields in the range of 5–20 μg/mL. The development of a continuous exchange cell‐free dialysis system (CECF) with a prolonged reaction time and freshly supplied reaction components was an important break‐through which significantly improved the total amount of synthesized proteins. Membrane protein yields in particular were raised to 100–700 μg/mL (Supplementary Fig. S1 and Table S1). Although a CECF reaction is approximately 10 times more expensive compared to a typical batch reaction an increase of the total protein yield up to 100‐fold was observed (Merk et al., 2015; Quast et al., 2016a). Therefore, it had proved possible to achieve a significant reduction of the total costs by a factor of 10 within the last years. The main costs of cell‐free synthesis systems arise through the addition of T7‐RNA‐polymerase, energy in the form of adenosine‐ and guanosine triphosphate and an energy regeneration system. At least the costs of exogenous added T7‐RNA‐polymerase might be decreased by in‐house‐production. The development of alternative energy‐rich components and the energy regeneration systems is already in process (Anderson et al., 2015). In summary, the increasing productivity of eukaryotic cell‐free systems, in combination with the reduced costs for lysates and the energy regeneration system are excellent preconditions to increase the yield‐on‐cost ratio significantly. Successful strategies to further optimize cell‐free synthesis of GPCRs and membrane proteins in general are mostly based on a variety of lipid structures like liposomes, micelles, bicelles, and nanodiscs (Bayburt and Sligar, 2010; Kalmbach et al., 2007; Lyukmanova et al., 2012). To date, several GPCRs, for example, endothelin A and endothelin B receptors (Proverbio et al., 2013) have been successfully synthesized using lipid structures in combination with cell‐free systems. However, just a few of them have also been investigated regarding their ligand binding (Arimitsu et al., 2014; Corin et al., 2011a; Ishihara et al., 2005; Klammt et al., 2011; Yang et al., 2011). Our study extends the list of cell‐free synthesized GPCRs by GRM1, GLP1R, GPR56, TSHR, CXCR4, CXCR5, and MOR, with the latter one being also investigated in the context of specific ligand binding properties. To further study, the mentioned GPCRs in detail it is of highly interest to demonstrate the synthesis of functional GPCRs in mg‐range. Therefore the used cell‐free system has to be scaled up. Up to date several efforts were investigated to establish cell‐free systems for a productive scale. The well‐known example of linearly scalable cell‐free system based on *E.coli* lysates produced 700 mg/L granulocyte‐macrophage colony‐stimulating factor in a volume up to 100 L (Zawada et al., 2011). Nevertheless the scalability of eukaryotic cell‐free systems has to be demonstrated. Moreover the high‐yield production in preparative scale of more complex proteins has to be further investigated, in particular, science sufficient amount of complex proteins are achieved in μL‐scale (Quast et al., 2016b).

Cell‐free systems based on insect cell lysates can produce correctly folded and functional proteins harboring posttranslational modifications (Dondapati et al., 2014; Sachse et al., 2012; Stech et al., 2014a; Zheng et al., 2014). However, first attempts in our study failed to detect specific ligand binding signals using cell‐free synthesized MOR. In this context, the addition of several detergents known to solubilise membrane structures (Corin et al., 2011b; Ishihara et al., 2005) was ineffective. Finally, fusion of melittin signal sequence to GPCR genes resolved this issue. This result is in line with previous studies where it was demonstrated that the insertion of the melittin signal sequence has improved the translocation and insertion efficiency of de novo synthesized proteins into microsomal vesicles (Brödel et al., 2013c; Stech et al., 2014b). Due to our results generated by SDS–PAGE, autoradiography and fluorescence microscopy, we assume that GPCRs are integrated in the vesicular membrane. This implicates, that a hydrophilic ligand, which is not able to cross a vesicular membrane, will most probably not reach the receptors binding pocket if this is located inside the vesicle. Using a radio‐ligand binding assay, it was demonstrated that cell‐free synthesized, melittin fused MOR was able to bind DAMGO. The effect of the melittin signal sequence is not fully understood. According to previous publications, demonstrating that the melittin signal sequence enhanced the translocation of secreted and type‐I‐transmembrane proteins into microsomes (Quast et al., 2015b), it can be assumed that this effect might also account for certain cell‐free synthesized GPCRs. In addition, fusion of a signal peptide (melittin signal sequence) to the target gene could have positive influence on the synthesis and orientation of the GPCR in the vesicular membrane (Köchl et al., 2002).

Taken together, our study underlines the potential of cell‐free protein synthesis systems for the synthesis of difficult to express proteins like GPCRs. Our results pave the way for future cell‐free production of GPCRs in sufficient amounts for downstream functional and structural analysis using eukaryotic cell‐extracts.

We thank Dipl.‐Ing. Doreen A. Wüstenhagen and Dipl. Nutritional Scientist Conny Mascher for the cultivation of *Sf*21 cells and the preparation of cell‐free extracts. We thank Nicole Vogel for technical assistance This work is supported by the German Ministry of Education and Research (BMBF, KMU‐innovativ: Biotechnologie—BioChance, No. 031A511; VIP0272, AZ 03V064).

## Supporting information

Additional supporting information may be found in the online version of this article at the publisher's web‐site.

**Figure S1**. Productivity of insect cell‐free systems. Yields of membrane proteins synthesized in insect cell‐free batch and dialysis systems are shown.
**Table S1**. Productivity of insect cell‐free systems for membrane protein synthesis.
