# Supplementary material for: Production of G protein‐coupled receptors in an insect‐based cell‐free system
Source: Biotechnol Bioeng. 2017 Jul 3;114(10):2328–38. doi: 10.1002/bit.26346 (PMC5599999; doi:10.1002/bit.26346)
Supplement: Supplementary file 1 — Figure S1. Productivity of insect cell‐free systems. Yields of membrane proteins synthesized in insect cell‐free batch and dialysis systems are shown. Table S1. Productivity of insect cell‐free systems for membrane protein synthesis. [file BIT-114-2328-s001.docx]

Figure S1: Productivity of insect cell-free systems. Yields of membrane proteins synthesized in insect cell-free batch and dialysis systems are shown. The development of dialysis systems significantly increased the yield of synthesized proteins. Triangles indicate cell-free synthesis reactions performed in dialysis mode.


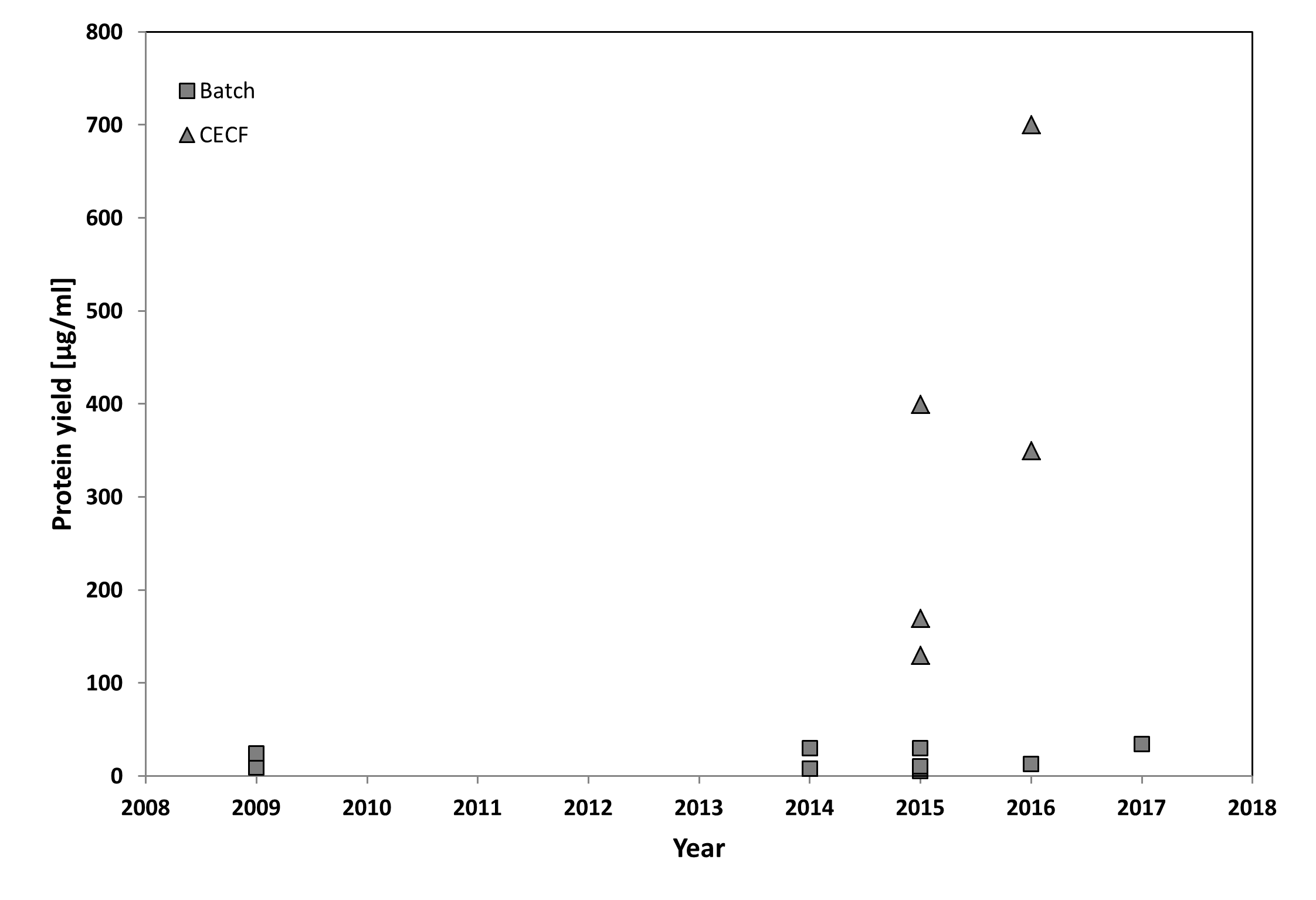


**Supplementary Table 1**: Productivity of insect cell-free systems for membrane protein synthesis

| Year | Protein yield [µg/ml] | Synthesis mode | Citation |
| --- | --- | --- | --- |
| 2009 | 9.2-23.9 | Batch | Kubick et al., 2009 |
| 2014 | 8  30 | Batch  Dialysis | Sachse et al., 2014; Dondapati et al., 2014  Stech et al., 2014 |
| 2015 | 5-30  130-700 | Batch  Dialysis | Quast et al., 2015; Merk et al., 2015  Merk et al., 2015 |
| 2016 | 13  300 | Batch  Dialysis | Quast et al., 2016  Quast et al., 2016 |
| 2017 | 34 | Batch | This publication |
